# Acute Renal Infarction Secondary to Atrial Fibrillation: A Rare Cause of Sudden Flank Pain

**DOI:** 10.7759/cureus.102387

**Published:** 2026-01-27

**Authors:** Asrar S Alshamrani, Rehab A Aljohani, Osamah A Alharbi, Osama F Almabadi, Anas E Ahmed

**Affiliations:** 1 Medicine, King Khalid University, Abha, SAU; 2 Medicine, Batterjee Medical College, Jeddah, SAU; 3 Medicine, Qassim University, Buraydah, SAU; 4 Medicine, Jeddah University, Jeddah, SAU; 5 Community Medicine, Jazan University, Jazan, SAU

**Keywords:** acute kidney injury, acute renal infarction, anticoagulation, atrial fibrillation, cardioembolic, contrast-enhanced ct, flank pain, renal ischemia, thromboembolism, wedge-shaped perfusion defect

## Abstract

Acute renal infarction is an uncommon and often underrecognized cause of sudden flank pain, posing diagnostic challenges due to its nonspecific presentation and laboratory findings. It may manifest with abrupt, severe flank discomfort, nausea, vomiting, and microscopic hematuria, frequently mimicking more common renal or musculoskeletal conditions. Risk factors such as atrial fibrillation significantly increase the likelihood of embolic events leading to segmental renal ischemia, particularly in patients with suboptimal anticoagulation. Imaging, especially contrast-enhanced computed tomography, is pivotal in establishing the diagnosis by identifying characteristic wedge-shaped perfusion defects, while supportive laboratory markers, including elevated lactate dehydrogenase and leukocytosis, may provide additional clues. Prompt recognition and initiation of anticoagulation therapy are central to preserving renal function and preventing further embolic complications. Clinical vigilance, timely diagnostic evaluation, and careful management can result in favorable outcomes, even in patients presenting with acute kidney injury, highlighting the importance of considering renal infarction in the differential diagnosis of sudden flank pain, particularly in those with cardiovascular risk factors.

## Introduction

Acute renal infarction is an uncommon and frequently underdiagnosed condition characterized by sudden cessation of blood flow to renal tissue, leading to ischemia and potential loss of renal function [1,2]. The condition often presents with nonspecific symptoms such as flank pain, nausea, vomiting, and hematuria, which can mimic more common disorders, including nephrolithiasis, pyelonephritis, or musculoskeletal pain [2,3]. Laboratory findings may reveal elevated lactate dehydrogenase (LDH), leukocytosis, and impaired renal function, but these markers are nonspecific [1-3]. Prompt recognition and timely management are critical to preserve renal function and prevent long-term complications. Cardiovascular disorders, particularly atrial fibrillation (AF), represent a significant risk factor for renal infarction due to the potential for cardioembolic events [3,4].

AF is a prevalent arrhythmia, especially among the elderly, and is associated with a heightened risk of thromboembolic complications, including stroke, mesenteric ischemia, and, less commonly, renal infarction [1-4]. Despite its rarity, acute renal infarction carries significant morbidity, and delays in diagnosis are common due to its nonspecific clinical presentation [3,4]. Imaging modalities, particularly contrast-enhanced computed tomography (CT), play a pivotal role in establishing the diagnosis [1-5]. Understanding the clinical features, risk factors, and management strategies for renal infarction in the context of AF is essential for improving patient outcomes and guiding appropriate anticoagulation therapy.

## Case presentation

A 68-year-old man with a past medical history significant for hypertension, type 2 diabetes mellitus, and paroxysmal AF, presented to the emergency department with a sudden onset of severe, left flank pain radiating to the groin, which began approximately six hours before admission. He described the pain as sharp, constant, and associated with nausea and one episode of nonbilious vomiting. The patient denied fever, dysuria, hematuria, recent trauma, or prior similar episodes. He reported adherence to his antihypertensive medications but admitted to inconsistent use of his oral anticoagulation due to concerns about bleeding risk. He had no history of kidney disease, recent travel, or urinary tract infections. His family history was notable for cardiovascular disease, and he was a former smoker with a 20-pack-year history, quitting 10 years ago.

On presentation, the patient was in acute distress due to pain. Vital signs revealed a blood pressure of 158/92 mmHg, a heart rate of 124 beats/minute with an irregular rhythm, a respiratory rate of 20 breaths/minute, an oxygen saturation of 97% on room air, and a temperature of 37.1°C. On general examination, he appeared uncomfortable, clutching his left flank. Cardiovascular examination confirmed an irregular rhythm with no murmurs, rubs, or gallops. Pulmonary auscultation was unremarkable. Abdominal examination revealed tenderness to palpation over the left costovertebral angle without rebound tenderness, guarding, or palpable masses. No lower limb edema or skin changes were noted. Neurological examination was nonfocal, and the remainder of the systemic examination was within normal limits.

Laboratory investigations demonstrated a serum creatinine of 1.8 mg/dL (baseline 1.0 mg/dL), blood urea nitrogen of 36 mg/dL, leukocytosis with white blood cell count 12 × 10⁹/L, mild elevation of LDH at 450 U/L, and normal liver function tests. Urinalysis showed trace proteinuria and microscopic hematuria without pyuria. Cardiac biomarkers, including troponin I, were within normal limits. Electrocardiography confirmed AF with a rapid ventricular response.

**Figure 1:**
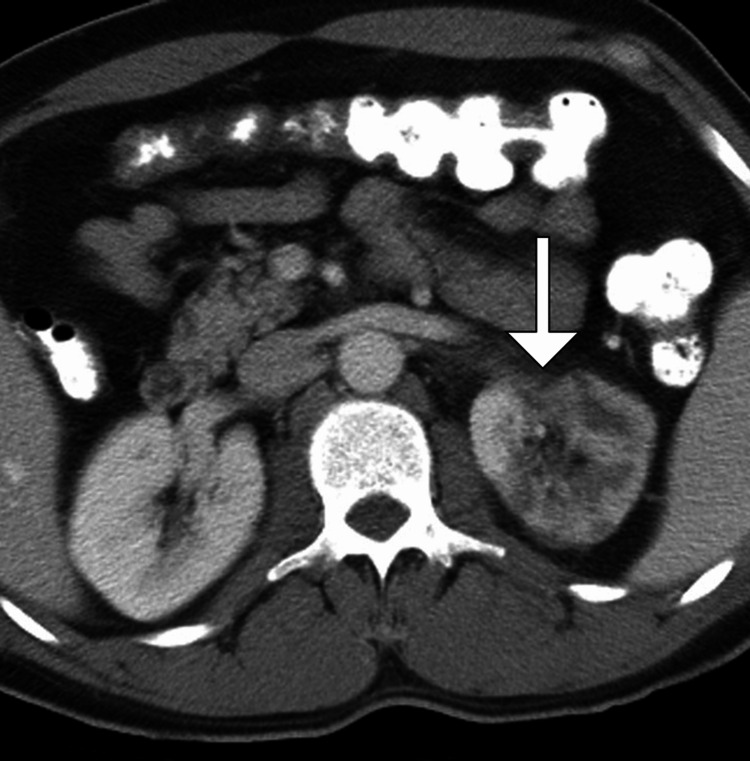
Axial contrast-enhanced CT image demonstrating a wedge-shaped area of decreased enhancement in the left kidney (arrow), consistent with an acute renal infarction CT: computed tomography

Given the acute flank pain with a history of AF and elevated LDH, contrast-enhanced CT of the abdomen and pelvis was performed, revealing a wedge-shaped area of decreased enhancement in the mid and lower pole of the left kidney, consistent with renal infarction. There was no evidence of obstructive uropathy, nephrolithiasis, or other intra-abdominal pathology (Figure 1).

Based on the clinical presentation, imaging findings, and history of AF, the differential diagnosis included renal infarction secondary to cardioembolic phenomena, renal artery thrombosis, pyelonephritis, nephrolithiasis, and musculoskeletal causes. The combination of acute flank pain, elevated LDH, hematuria, irregular heart rhythm, and imaging findings strongly supported a diagnosis of acute renal infarction due to embolic occlusion in the context of AF.

The patient was admitted to the hospital and started on intravenous (IV) hydration and analgesia with opioids for pain control. Anticoagulation therapy was initiated with IV unfractionated heparin, followed by transition to oral warfarin once renal function stabilized. Blood pressure and heart rate were carefully monitored, and rate control for AF was achieved using IV diltiazem. Renal function was closely followed, and repeat serum creatinine remained stable. No invasive interventions were required as there was no evidence of ongoing ischemia or hemodynamic instability.

During hospitalization, the patient’s symptoms gradually improved, with resolution of flank pain by day 4. He remained hemodynamically stable, with normalization of inflammatory markers. A transthoracic echocardiogram revealed normal left ventricular function and no intracardiac thrombus, supporting the diagnosis of embolic renal infarction secondary to AF. The patient was discharged on day 6 with instructions to continue oral anticoagulation, optimize blood pressure and diabetes control, and follow up in the nephrology and cardiology clinics. At one-month follow-up, the patient remained asymptomatic, with stable renal function and well-controlled heart rate.

## Discussion

Acute renal infarction is a rare but potentially serious condition that often remains underdiagnosed due to its nonspecific clinical presentation [1,2]. The incidence has been reported as 0.007%-1.4% in autopsy and hospital-based studies, highlighting both its rarity and the likelihood of missed diagnoses [3,4]. The most common etiologies include cardioembolic events, thrombotic occlusions, and, less frequently, vascular dissections or hypercoagulable states [5,6]. Among these, AF represents a major risk factor, accounting for up to 50% of cardioembolic renal infarctions [1-4]. In this case, the patient’s paroxysmal AF, coupled with suboptimal anticoagulation adherence, likely precipitated an embolic occlusion of a segmental renal artery, resulting in ischemia and acute renal injury.

The clinical manifestations of renal infarction are typically nonspecific and often mimic more common conditions, such as nephrolithiasis, pyelonephritis, or musculoskeletal pain [3,4]. Flank pain is the most frequently reported symptom, often accompanied by nausea, vomiting, and occasionally hematuria. Laboratory findings are likewise nonspecific; elevated LDH, leukocytosis, and mild renal function derangements are common but not pathognomonic [1-4]. Imaging studies, particularly contrast-enhanced CT, remain the diagnostic gold standard, revealing wedge-shaped perfusion defects corresponding to areas of ischemia, as seen in our patient. Doppler ultrasonography and magnetic resonance imaging can serve as adjuncts but are less sensitive in acute settings [3,4].

Management strategies for acute renal infarction hinge on early recognition and prompt restoration of renal perfusion when feasible, alongside prevention of further thromboembolic events [1,5]. Anticoagulation is the cornerstone of therapy in cases secondary to AF, with heparin bridging followed by long-term oral anticoagulants, as was successfully implemented in this patient. The decision for interventional procedures, such as catheter-directed thrombolysis or surgical embolectomy, is reserved for cases with bilateral involvement, solitary kidneys, hemodynamic instability, or evidence of ongoing ischemia. Supportive care, including hydration, pain management, and close monitoring of renal function, remains critical in all cases [1-6].

This case underscores several important clinical lessons. First, clinicians must maintain a high index of suspicion for renal infarction in patients presenting with acute flank pain, particularly in the presence of AF or other embolic risk factors. Second, early imaging and laboratory evaluation, including LDH and renal function tests, can expedite diagnosis and improve outcomes. Third, adherence to anticoagulation therapy in patients with AF is essential not only for stroke prevention but also to mitigate the risk of systemic embolic events, including renal infarction. Finally, a comprehensive follow-up is vital to monitor renal recovery and optimize long-term cardiovascular risk management.

## Conclusions

Acute renal infarction, although rare, should be considered in any patient presenting with sudden flank pain, particularly in the context of AF or other embolic risk factors. Early recognition, timely imaging, and prompt initiation of anticoagulation are essential to preserve renal function and prevent further systemic embolic events. This case highlights the importance of maintaining a high index of suspicion, ensuring adherence to anticoagulation therapy, and providing vigilant follow-up, underscoring that heightened clinical awareness can significantly improve patient outcomes in this underdiagnosed but potentially serious condition.
